# Atlantic origin of asynchronous European interdecadal hydroclimate variability

**DOI:** 10.1038/s41598-019-47428-6

**Published:** 2019-07-29

**Authors:** Davide Zanchettin, Thomas Toniazzo, Carla Taricco, Sara Rubinetti, Angelo Rubino, Nazario Tartaglione

**Affiliations:** 1 University Ca’Foscari of Venice, Dept. of Environmental Sciences, Informatics and Statistics, Via Torino 155, 30172 Mestre Venezia, Italy; 2 NORCE Norwegian Research Centre, Bjerknes Centre for Climate Research, Jahnebakken 5, 5007 Bergen, Norway; 3 University of Turin, Dep. of Physics, Via Pietro Giuria 1, 10125 Torino, Italy; 4 Istituto Nazionale di Astrofisica, Osservatorio Astrofisico di Torino (OATo-INAF), Strada Osservatorio 20, 10025 Pino Torinese, Italy

**Keywords:** Hydrology, Projection and prediction, Atmospheric science, Climate-change impacts

## Abstract

Discharge time series of major large-catchment European rivers are known to display significant decadal and interdecadal fluctuations. However, the hydroclimate variability causing such fluctuations remains poorly understood, particularly due to a lack of a spatio-temporal integrated assessment. Here, we demonstrate for the first time that European hydroclimate variability is dominated by a meridional delayed oscillation characterized by a lag of approximately 5 years in interdecadal discharge fluctuations of continental (northern) European rivers with respect to those of Euro-Mediterranean (southern) rivers. We demonstrate a connection of this coherent signal with the large-scale atmospheric circulation over the North Atlantic, and suggest a hitherto unexplored multiannual atmosphere-ocean mechanism in the subpolar North Atlantic at its root.

## Introduction

**Figure 1:**
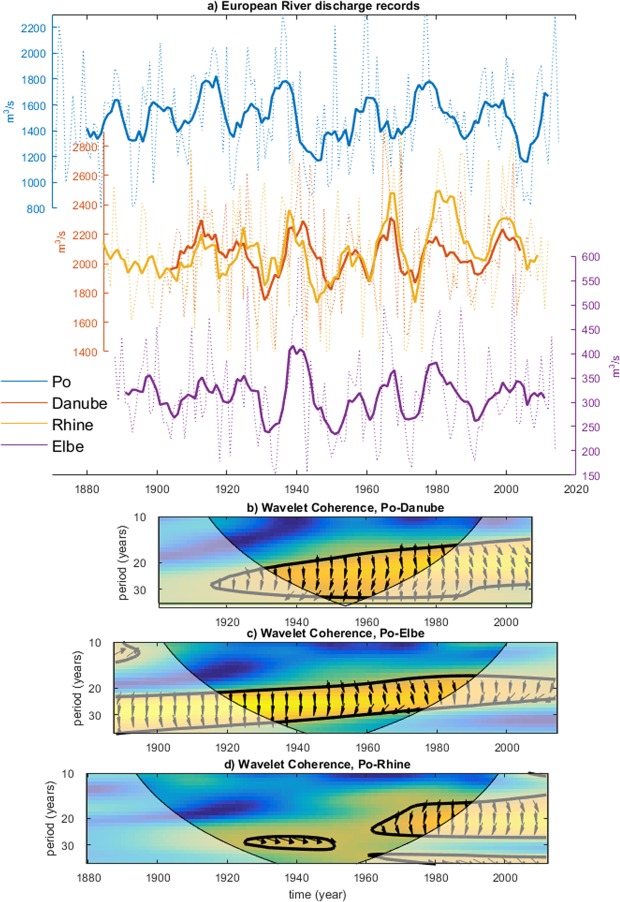
Observed asynchronous bi-decadal fluctuations of major European river discharges. (**a**) Annual mean, smoothed (7-year running mean) discharge data of the Po at Pontelagoscuro (blue), Danube at Bratislava (dark orange), Rhine at Koeln (mustard) and Elbe at Decin (violet). Dashed lines: raw annual mean time series. (**b**–**d**) Wavelet coherence spectra between annual-average European river discharge data. Thick contours identify the 5% significance level. Arrows identify the phases (eastward-pointed arrows identify co-phase, Po discharges lead for clockwise rotation from the co-phase axis). The shaded region indicates where edge effects occur.

Observed discharge time series of major European rivers are characterized by significant variability in the interdecadal time scales (10–30 years)^1–6^. Figure 1a shows representative time-series of four large-catchment European rivers, i.e. the Po, the Danube, the Rhine and the Elbe. The presence of large interdecadal discharge fluctuations is not specific to these rivers (Supplementary Fig. S1), and a quantification of the interdecadal fraction of the total river discharge variability (Supplementary Fig. S2) confirms the visual impression from the time-series, with variances ranging between one fifth and one third. Some rivers, most noticeably the Po and the Danube, show a seasonal separation between the dominant periodicity within the range of interdecadal time scales, with decadal fluctuations most prominent during the summer semester and bidecadal fluctuations during the winter semester (Supplementary Fig. S3). Similar interdecadal variability has been observed in European precipitation and linked to large-scale atmospheric and oceanic circulation anomalies in the North Atlantic^7,8^. Analysis of individual rivers has revealed that the connection between European hydroclimate variability and North Atlantic climate can be non-stationary^9^ and affected by temporal lags^10^. But such relationships need not be uniform within Europe.

A comparative analysis between selected discharge series of large-catchment rivers north and south of the Alps reveals that bidecadal fluctuations (i.e., the portion of interdecadal variability around the ~20-year timescale) in the Po River - representative of Italian Alpine rivers^6^ as well as Dinaric Alpine rivers (Supplementary Fig. S4) - lead those in the Danube, Rhine and Elbe Rivers - representative of continental rivers (Fig. 1b–d). The observed phase-lag relationship is most apparent after 1920, when interdecadal fluctuations with a timescale of about 20 years appear more prominently as a near-regular oscillation (Fig. 1a). The wavelet coherence spectra in Fig. 1b–d confirm the robustness of this phase-lag relationship in recent decades. Arrows pointing in the direction opposite to the y-axis indicate that the Po leads in quadrature, which, for periodicities of about 20 years, correspond to a leading time of about 5 years. Such a lead time emerges also robustly from cross-correlation analysis (Supplementary Fig. S5).

Runoff data from meteorological reanalysis^11^ capture the prominent interdecadal evolution of observed Mediterranean and continental river discharges as well as their associated phase lag (Supplementary Figs S6 and S7). Reanalysis data can thus aid in the interpretation of the observed bidecadal discharge evolutions of major European rivers. The reanalysis shows very similar spatiotemporal characteristics between decadal-scale anomalies in runoff and precipitation (Supplementary Figs S7–S9). Reanalyzed evaporation and evapotranspiration contributions, often critically affecting the precipitation-discharge relationship^3,12^, exert an overall stronger contribution in summer compared to winter, although patterns are patchier than for precipitation (Supplementary Fig. S10). The lagged relationship seen between discharge records from continental and Mediterranean rivers thus reflects only partly a lagged relationship between regional precipitation north and south of the Alps, i.e., it originates from hydroclimate dynamics governing precipitation (providing the inflow to the riverine systems) as well as from runoff processes (converting precipitation into discharge).

**Figure 2:**
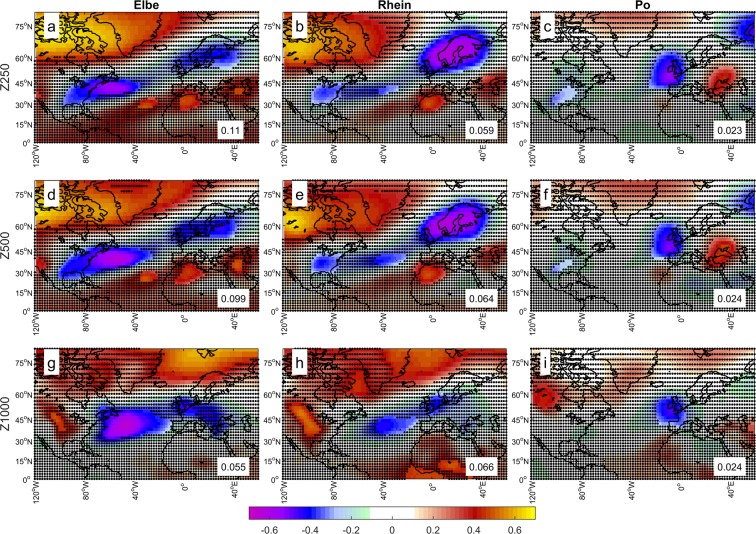
Large-scale atmospheric patterns associated to interdecadal variability of European river discharges. Correlation between winter (October-March average) geopotential heights at 250 hPa (Z250), 500 hPa (Z500) and 1000 hPa (Z1000) and major European river discharges. Significance threshold at p = 0.1. The numbers are area-weighted mean squared errors with the NAO correlation pattern for the same period. Data are band-pass filtered (9–50 years) and linearly detrended before the analysis.

Wintertime decadal-scale variations of European river discharges are robustly associated with significant large-scale atmospheric circulation anomalies (Fig. 2). No such relationships exist for the summer semester (not shown). For the Po river, interdecadal variations in wintertime discharge are associated with changes of the meridional wind over central Europe and a dipolar large-scale geopotential pattern with a low-pressure center over the Celtic Sea and the Gulf of Biscay and a high-pressure center over the Black Sea (Fig. 2c,f,i). This pattern is consistent with the main pattern obtained by Dünkeloh and Jacobeit^2^ for the joint variability of North Atlantic-European large-scale atmospheric circulation and Mediterranean precipitation. A low-pressure system over the Bay of Biscay is typically linked with intense events over the Piedmont region, where the Po river has its source^13–15^. By contrast, the large-scale winter atmospheric circulation patterns associated with interdecadal variability of continental river discharges features a ridge-trough-ridge system extending across the North Atlantic, with centers forming a quadrupole around low-pressure centers located over Scandinavia and the Gulf Stream separation region, and high-pressure centers located over north-western Africa and Greenland (Fig. 2a,b,d,e,g,h). These patterns only partly project on the North Atlantic Oscillation (NAO) pattern, which is typically associated with interannual-to-decadal European river discharge variability^3–5^.

The evidence presented above leads us to hypothesize that the 5-year delay identified between decadal discharge variations in south- and north-European rivers is linked to a multiannual north-eastward displacement of the wintertime European low pressure center from the Bay of Biscay to Scandinavia (Fig. 2e,f). To test this hypothesis, we perform a composite analysis of seasonal atmospheric and oceanic anomalies based on peak wet and peak dry phases in the interdecadal evolution of Po river discharge. We test the significance of the composite circulation anomalies for different lags by using a statistical bootstrap method which accounts for the uncertainty in the identification of the reference peak wet and dry years.

**Figure 3:**
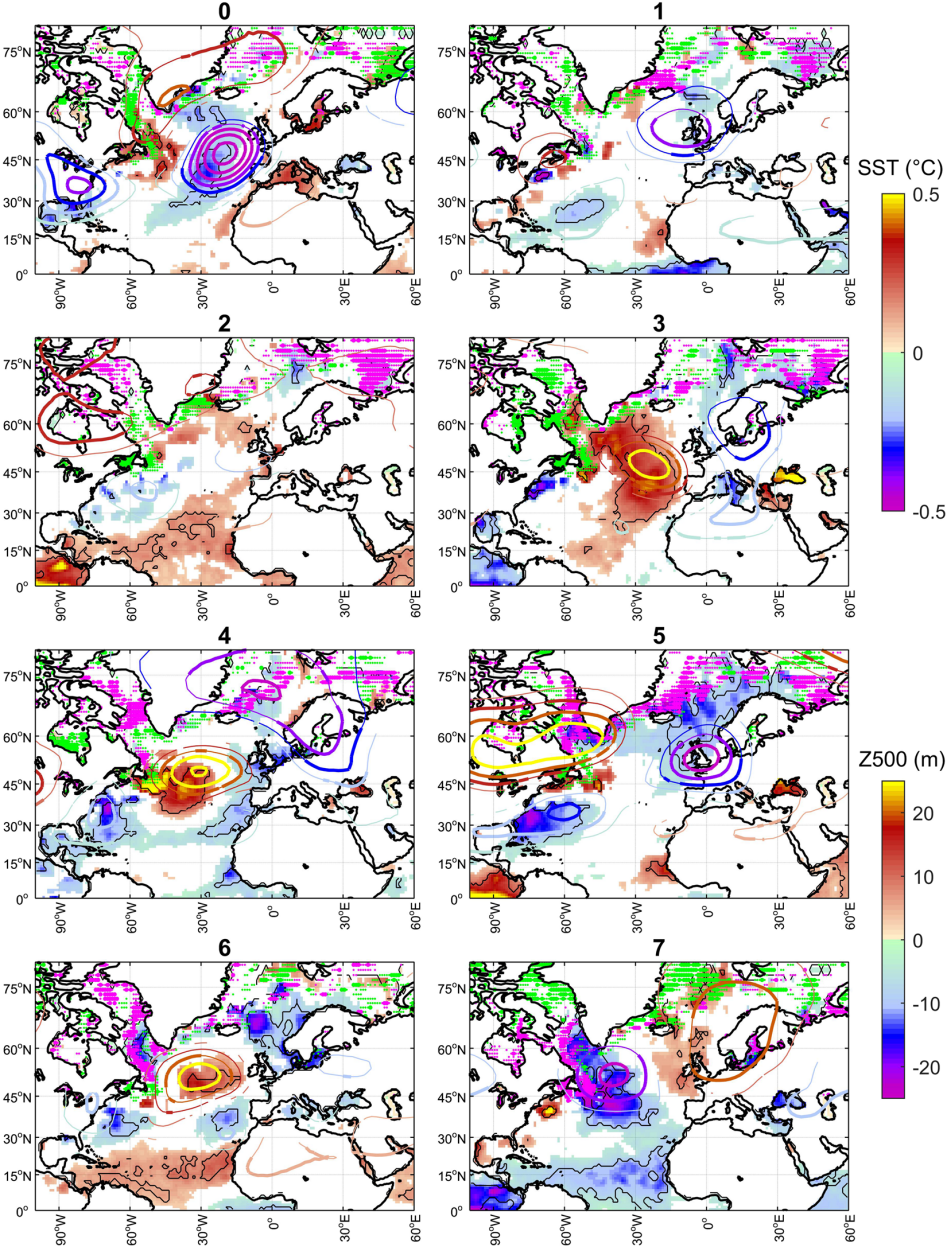
Multiannual evolution of anomalous winter patterns around peak wet phases linked to decadal variability of Po River discharges. Shading: winter (Oct-Mar) sea-surface temperatures, only for significant signals at 75% confidence (black contours identify region significant at 90% confidence); dots: March sea ice concentration, only for local increases (magenta) and decreases (green) significant at 75% (small) and 90% (large) confidence; contour: 500 hPa OM geopotential height, only for significant values at 75% (thin) and 90% (thick) confidence. Z500 data are linearly detrended, sea-surface temperature and sea-ice data are detrended with a second order polynomial fit to the data. Results are for years {1902 1914 1936 1950 1964 1978 1996} accounting for uncertain sampling. Panel titles indicate the lag, in years, from the reference years. Sea ice results are only indicative, given the more limited temporal coverage of the dataset.

During peak discharge phases of the Po river, winter-time low-pressure anomalies over the eastern North Atlantic are associated with significant sea-surface warming and sea ice reduction in the Labrador Sea (Fig. 3, lag 0). A cold anomaly is found in the central North Atlantic, partly along the low-pressure trough, and partly extending to the north-eastern edge of the subpolar gyre. The subpolar gyre warms considerably in the subsequent years (Fig. 3, lags 1–3), and the sea-surface temperature (SST) anomaly pattern evolves toward a tri-polar structure with a warm center at the intergyre boundary, and cold centers in the subtropical western North Atlantic and in the Nordic Seas (Fig. 3, lags 4–5). Sea-ice cover increases significantly over the Barents Sea and more generally along the sea-ice edge in the Nordic Seas. At a lag of 5 years, cold conditions are generally prevalent and a tripolar structure in 500 hPa geopotential height emerges, which corresponds to the correlation pattern associated with high discharge from continental rivers (Fig. 2d,e). The height anomaly pattern appears to be part of a wave-train connected with the North Pacific that projects onto the Pacific North American pattern. Later (lag 6), SSTs cool and sea-ice cover grows in the Labrador Sea while the Nordic Seas tend to warm, and the sea ice recedes across the Arctic Ocean. These patterns and the timescale of their evolution broadly match a mode of variability in the North Atlantic identified in several modelling studies^16–18^. Although there is no agreement in the details of the forcing mechanism and timescale across different models^16–19^, this mode is associated with a damped internal oscillation arising from NAO-ocean circulation interactions centered in the midlatitudes. The anomalies in surface wind-stress curl at lag 5 years bear resemblance with the corresponding pattern identified by Schneider and Fan^17^. In addition to midlatitude processes, the prominent high-latitude anomalies suggest that the multiannual mechanism may also involve interactions between Arctic sea ice, freshwater export from the Arctic and the subpolar gyre circulation^20–22^.

In summer, aspects of the development of oceanic anomalies after peak wet phases linked to interdecadal variability of Po discharges resemble the winter mechanism, including an interannual warm-to-cold transition of the Labrador Sea, temporary warming of the subpolar gyre and significant cooling in the Nordic Seas around lag of five years (Supplementary Fig. S11). However, atmospheric anomalies remain largely nonsignificant. This seasonal asymmetry between winter and summer observed in the interdecadal evolution of North Atlantic and European climates agrees with recent findings based on climate model simulations^22^. It reflects the tighter coupling between ocean and atmosphere in the winter season compared to the summer season, and further points toward high-latitude feedbacks being potentially relevant for the mechanism underlying the lag.

**Figure 4:**
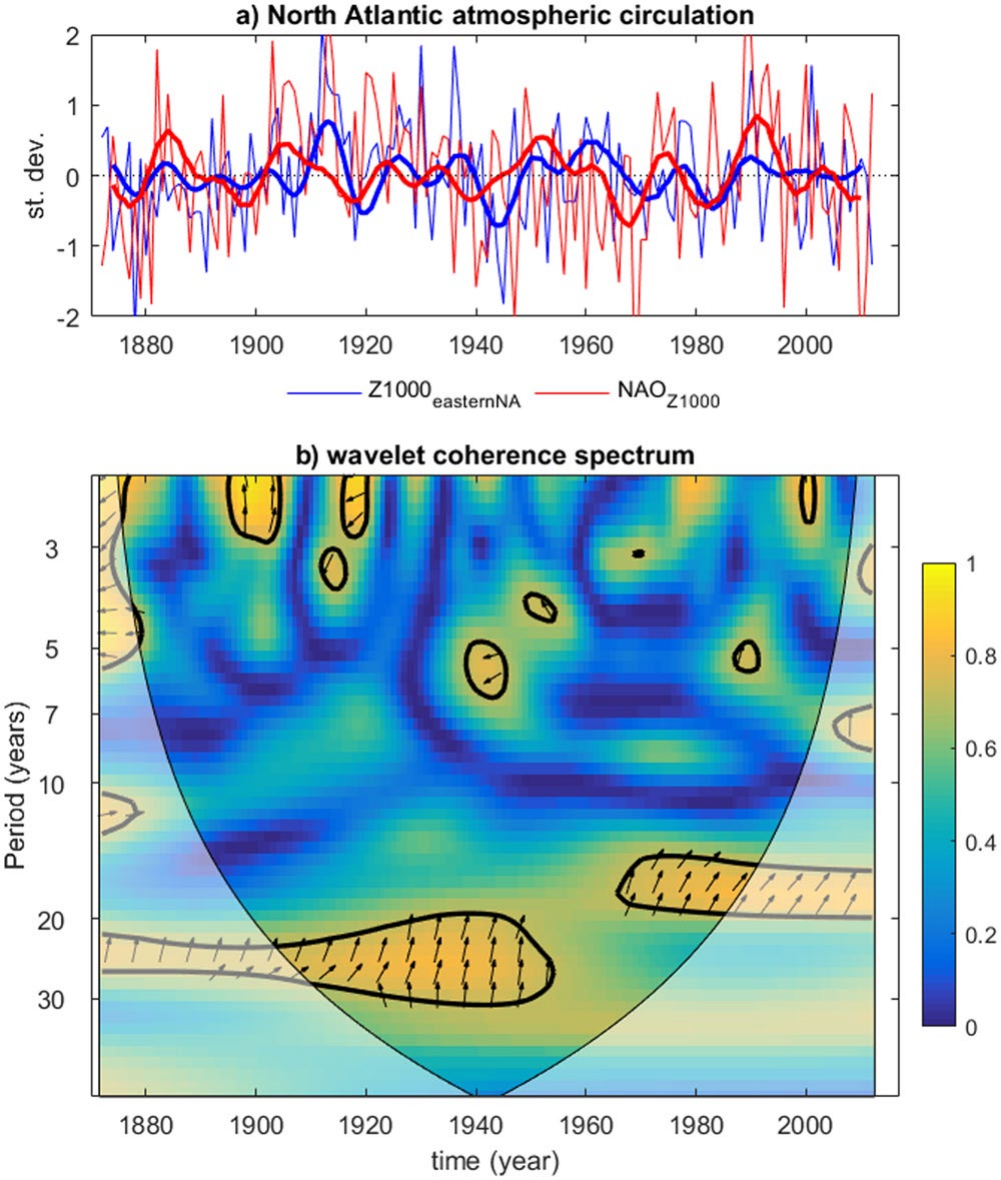
Asynchronous interdecadal fluctuations in North Atlantic Oscillation (red) and residual atmospheric pressure variability over the eastern North Atlantic (blue). (**a**) Winter (October-March) average time series (thin lines: raw data; thick lines: 9–50-year bandpass filtered data). (**b**) Wavelet coherence spectrum between the raw data. Thick contours identify the 5% significance level. Arrows identify the phases (eastward-pointed arrows identify co-phase, data from the Bay of Biscay region lead for clockwise rotation from the co-phase axis). The Eastern Atlantic index is defined as the residual of the linear regression of the Z1000 NAO index on the field-average Z1000 values in the domain 15–0°W longitude and 40–50°N latitude. All data linearly detrended before analysis.

The large-scale circulation anomalies associated with dry phases of Po discharges (Supplementary Figs S12 and S13) reflect an appreciable degree of symmetry with those composited for the wet phases, such as the transition from cold to warm SST anomalies in the Nordic Seas together with cooling of the subpolar gyre and along the Gulf Stream. A possible explanation of the weaker atmospheric signals is the longer duration of the decadal dry phases observed for the Po, with associated larger uncertainty in the identification of their peak. However, we note that by our selection, peak-wet years lag peak-dry years by about 7 years. Accordingly, in our analysis a strong negative pressure anomaly sits over the eastern North Atlantic at lag 7 (Supplementary Fig. S12), which superposes well with the lag-0 pattern observed in Fig. 3. As revealed by wavelet coherence analysis, there is a significant phase-lagged interdecadal variability linking NAO variations and residual atmospheric pressure variability over the eastern North Atlantic (Fig. 4), with the NAO leading by a few years as also confirmed by cross-correlations (not shown). Therefore, the NAO appears to lag as well as to lead (Fig. 4) the anomalous atmospheric conditions over the mid-latitude eastern North Atlantic. The alternation of decadal wet and dry phases in European hydroclimates and the associated meridional delayed oscillation appear thus to be associated with a continuous interaction between NAO and the ocean-sea ice-atmosphere coupled variability in the subpolar North Atlantic. Furthermore, the domain of the proposed mechanism partly superposes on that of the Atlantic Multidecadal Oscillation (AMO) describing multidecadal variations of North Atlantic sea-surface temperature^23,24^ and known to affect European hydroclimates^7,10^. Thus, the multi-annual mechanism that we suggest to be responsible for the lagged co-variability of European hydroclimate in different regions is not intended to invalidate previously identified teleconnections between interdecadal fluctuation of European river discharges and large-scale modes of variability in the Atlantic sector. Instead, we suggest that the paradigms of such climate modes as the NAO and the AMO be considered in a wider context where they form part of the observed co-variability over a larger North-Atlantic and pan-European region.

Our results provide a new opportunity to improve water management: Europe has abundant water resources, yet it is a water stress continent. Pro capita water availability is an actual issue in Europe especially in Mediterranean Countries, where a high water demand for irrigated agriculture, tourism, energy production and provision of drinking water must reconcile with water scarcity and droughts^25^. Severe and extensive droughts can impact Central-Northern Europe as well, as exemplified by the drought of summer 2018^26^. Parts of Europe must also face recurrent flooding and the social and economic losses it brings^27^. Climate change may exacerbate this stressed hydroclimatic situation as future climate warming scenarios indicate an asymmetric continental precipitation response to increased anthropogenic greenhouse gas emissions with wetting over Northern-Central (or continental) Europe and drying over the Mediterranean region^28^. Our results show prominent interdecadal river discharge variability in contrast to only sporadic significant long-term trends (Supplementary Fig. S2 and Table S1). The dominance of interdecadal variability over long-term trends motivates a strengthened focus on the assessment and prediction of European decadal climate variability. In this regard, improved predictions can be achieved based on improved understanding and simulation of the feedbacks between large-scale atmospheric circulation, the subpolar gyre and sea ice in the North Atlantic and Artic oceans, whose phasing is set by interdecadal changes of anomalous atmospheric pressure over the eastern North Atlantic. Current climate models are known to poorly simulate different aspects of observed and reconstructed decadal hydroclimate variability^29,30^. A preliminary assessment on a multi-model ensemble of historical simulations suggests that most climate models do not produce significant interdecadal winter precipitation variability in different European regions nor multiannual phase delays between continental and Euro-Mediterranean precipitation compatible with the observed features illustrated here (Supplementary Figs S14 and S15). Understanding the reasons behind this general lack of skills could foster progress toward improved climate models and prepare the ground to test the robustness of the mechanism proposed here.

## Data and Methods

River discharge data are from the Global Runoff Data Centre, 56058 Koblenz, Germany. Po River discharge data are those described in ref.^12^, which include reconstructed values before 1917, updated to 2015 using values provided by ARPA-Veneto.

Reanalysis data are from the monthly NOAA-CIRES 20th Century Reanalysis V2 dataset^11^. Support for the Twentieth Century Reanalysis Project dataset is provided by the U.S. Department of Energy, Office of Science Innovative and Novel Computational Impact on Theory and Experiment (DOE INCITE) program, and Office of Biological and Environmental Research (BER), and by the National Oceanic and Atmospheric Administration Climate Program Office.

Wavelet coherence spectra are calculated using the cross wavelet and wavelet coherence package by Aslak Grinsted, John Moore and Svetlana Jevrejeva^31,32^.

Temporal uncertainty in the sampling of anomalous wet and dry phases (ref. Figs 3 and S9–S11) is accounted as follows. Given a set of *n* peak anomaly years Y_1,..,n_, we generate *m* sets of random *n* integer values V_m;1,…,n_ obtained by rounding samples from the normal distribution N(0,1); we then sum Y and V to obtain *m* realizations of uncertain peak anomaly years.

## Supplementary information

Supplementary Material
