# Supplementary material for: Atlantic origin of asynchronous European interdecadal hydroclimate variability
Source: Sci Rep. 2019 Jul 29;9:10998. doi: 10.1038/s41598-019-47428-6 (PMC6662824; doi:10.1038/s41598-019-47428-6)
Supplement: Supplementary file 1 — Supplementary Material [file 41598_2019_47428_MOESM1_ESM.docx]

**Atlantic origin of asynchronous European interdecadal hydroclimate variability**

Davide Zanchettin, Thomas Toniazzo, Carla Taricco, Sara Rubinetti, Angelo Rubino, Nazario Tartaglione

**SUPPLEMENTARY MATERIAL**

**Supplementary Figure S1** – Time series of major European river discharges. (a) Annual-mean time series; (b) standardized time series. Data smoothed with a 7-year running mean filter. The discharge series used in the main study are shown with a thick line (color code as for Figure 1).

**Supplementary Figure S2** – Fraction (in percent) of total variance of major European river discharges explained by interdecadal fluctuations in the range of 9-25 years, for the annual, winter and summer average time series. Note, the considered range of interdecadal fluctuations excludes variability at O(8 years) which is significant in winter in certain rivers.


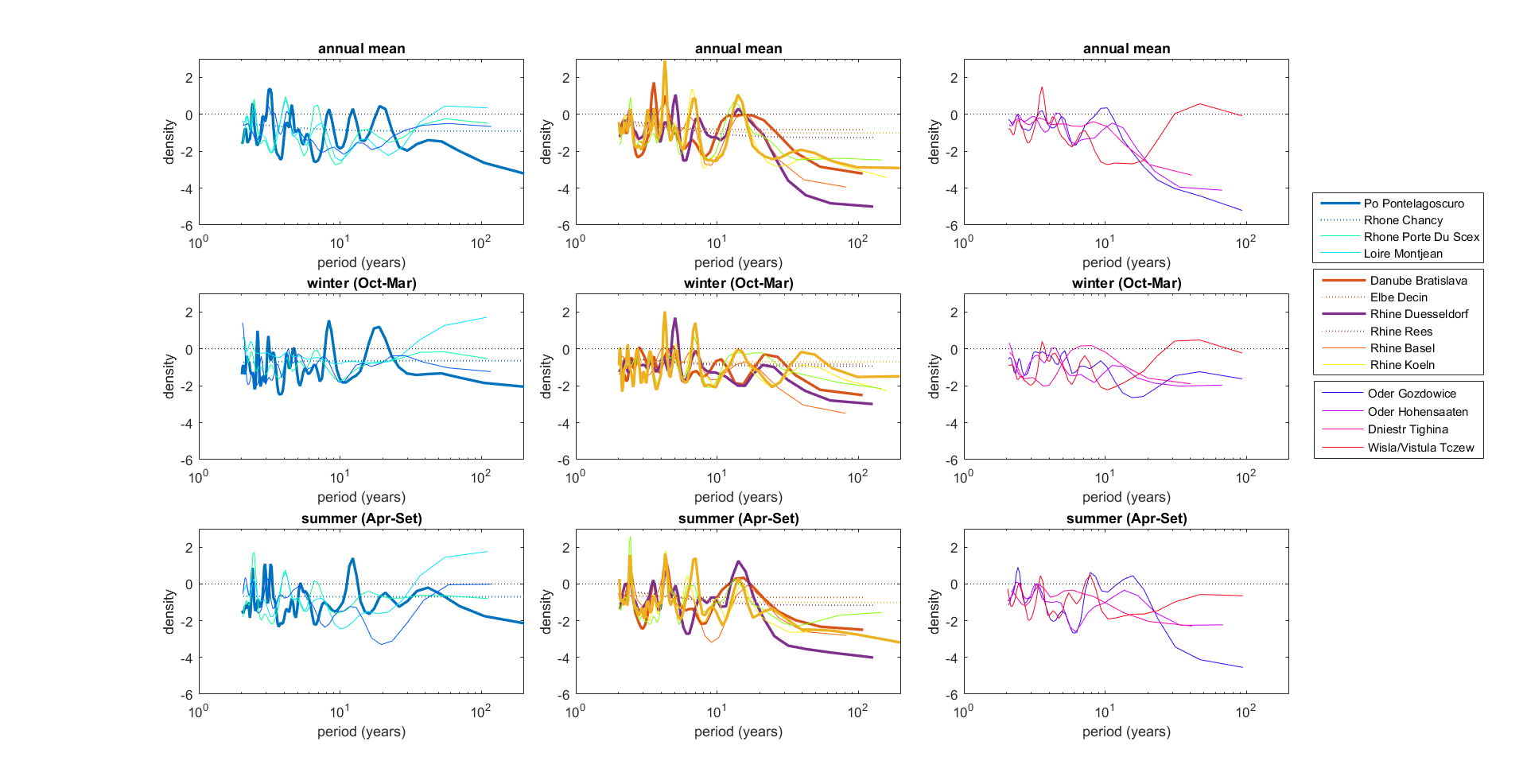


**Supplementary Figure S3** - Density spectra of standardized (a) annual, (b) winter (October-March) and (c) summer (April-September) average discharge time series of major European rivers relative to their 95% confidence level (horizontal black dotted line). The discharge series used in the main study are shown with a thick line and the corresponding 90% confidence level is also shown as dotted line (color code as for Figure 1). Note that the different discharge time series cover different periods, which affects the comparability of the spectra.

**Supplementary Figure S4** – Comparison between interdecadal discharge fluctuations of the Po River and other rivers originating in the Dinaric Alps. (top) Annual-mean time series; (middle) winter (October-March); (bottom) summer (April-September). Data are smoothed with a 7-year running mean filter and standardized. Only annual-average data of Adige, Brenta and Piave are available.

**Supplementary Figure S5** – Cross-correlation between smoothed (7-year running mean) annual time series of Po River discharges and Elbe, Danube and Rhine river discharges. Stars mark correlations that are statistically significant (p=0.05) accounting for autocorrelation in the series. Note that the maximum absolute correlation is observed at lag -5, i.e., when the Po discharge leads the other river discharges.

**Supplementary Figure S6** – Comparison between observed discharges (blue) and reanalyzed runoff (red) for the winter (October-March average, om) and summer (April-September average, as) season for three representative large-catchment European rivers. Spatial averages of runoff are calculated over the domain 9-14°E, 44-47°N for the Po, 4-9°E, 47-53°N for the Rhine and 10-15°E, 50-54°N for the Elbe. The shown time series are band-pass filtered (9-50 years), linearly detrended and standardized. Full-period correlation statistics (r) with associated p-value (in parentheses) are shown for both the raw, unfiltered data and the band-pass filtered data. Note the overall better agreement for the winter semester compared to the summer semester, and the better agreement for Po and Elbe compared to Rhine.


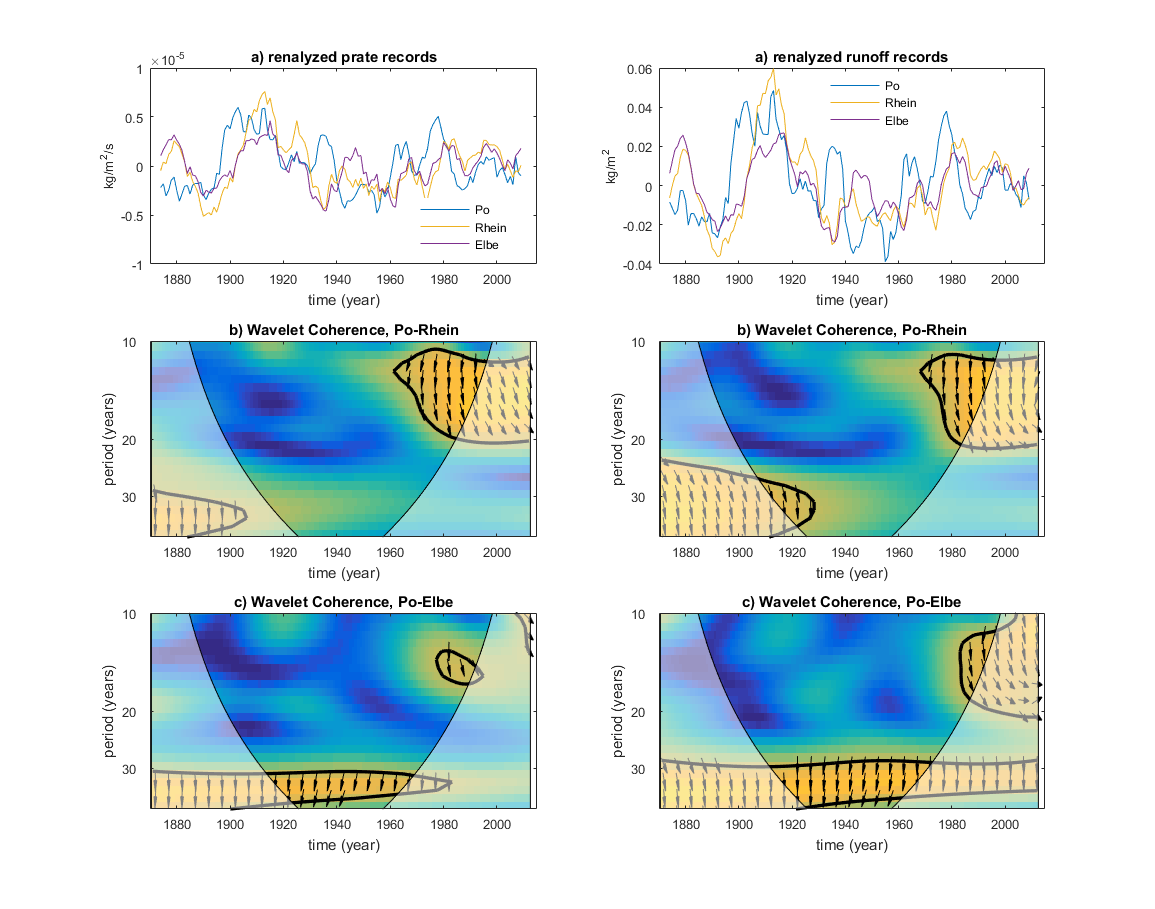


**Supplementary Figure S7** – Asynchronous interdecadal fluctuations in reanalyzed precipitation and runoff data associate to major European rivers. Spatial averages are calculated over the domain 9-14°E, 44-47°N for the Po, 4-9°E, 47-53°N for the Rhine and 10-15°E, 50-54°N for the Elbe. (**a,d**) Annual mean, smoothed (7-year running mean) precipitation (prate) and runoff data of Po, Rhine and Elbe rivers. (**b**,**c,e,f**) Wavelet coherence spectra between annual-average precipitation and runoff data of different rivers. Thick contours identify the 5% significance level. Arrows identify the phases (eastward-pointed arrows identify co-phase, Po discharges lead for clockwise rotation from the co-phase axis). The shaded region indicates where edge effects occur.

**Supplementary Figure S8** – Correlation of (a-c) winter (October-March average) and (d-f) summer (April-September average) reanalyzed runoff with observed large-catchment European river discharges (a,d: Elbe; b,e: Rhine; c,f: Po). Dots mark grid points where the correlation is statistically not significant (p>0.1) accounting for autocorrelation. Data are band-pass filtered (9-50 years) and linearly detrended before the analysis. The correlations of gridded reanalyzed runoff with the discharge data clearly reflect the catchments of interest, with an evident separation especially in the winter semester.


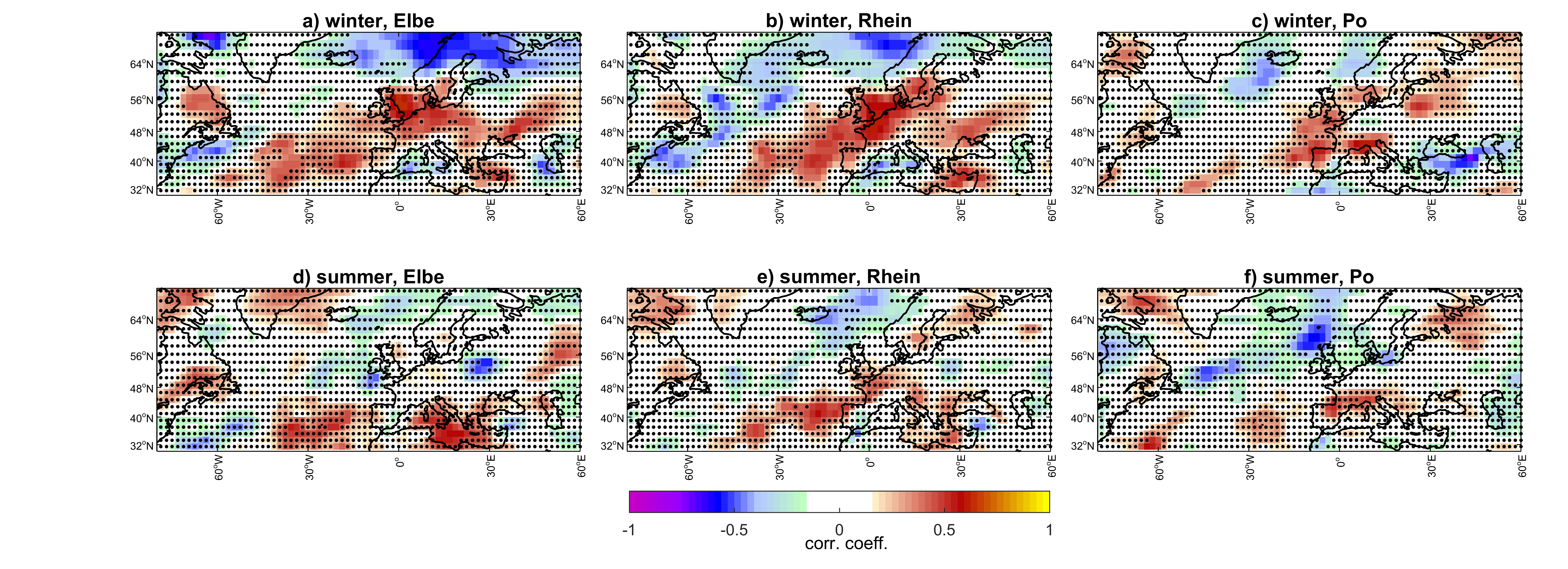


**Supplementary Figure S9** – Correlation of (a-c) winter (October-March average) and (d-f) summer (April-September average) reanalyzed precipitation with observed large-catchment European river discharges (a,d: Elbe; b,e: Rhine; c,f: Po). Dots mark grid points where the correlation is statistically not significant (p>0.1) accounting for autocorrelation. Data are band-pass filtered (9-50 years) and linearly detrended before the analysis. As for runoff, the correlations of gridded reanalyzed precipitation with the discharge data clearly reflect the catchments of interest, with an evident separation especially in the winter semester.


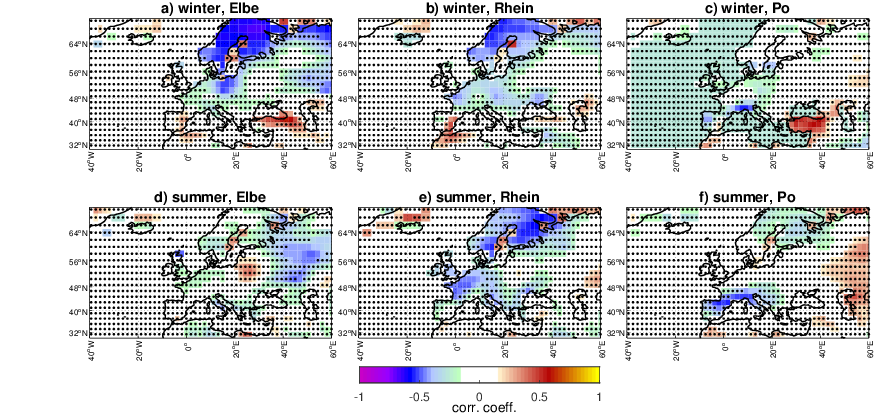


**Supplementary Figure S10** – Correlation of (a-c) winter (October-March average) and (d-f) summer (April-September average) reanalyzed potential evaporation plus transpiration with observed large-catchment European river discharges (a,d: Elbe; b,e: Rhine; c,f: Po). Dots mark grid points where the correlation is statistically not significant (p>0.1) accounting for autocorrelation. Data are band-pass filtered (9-50 years) and linearly detrended before the analysis.


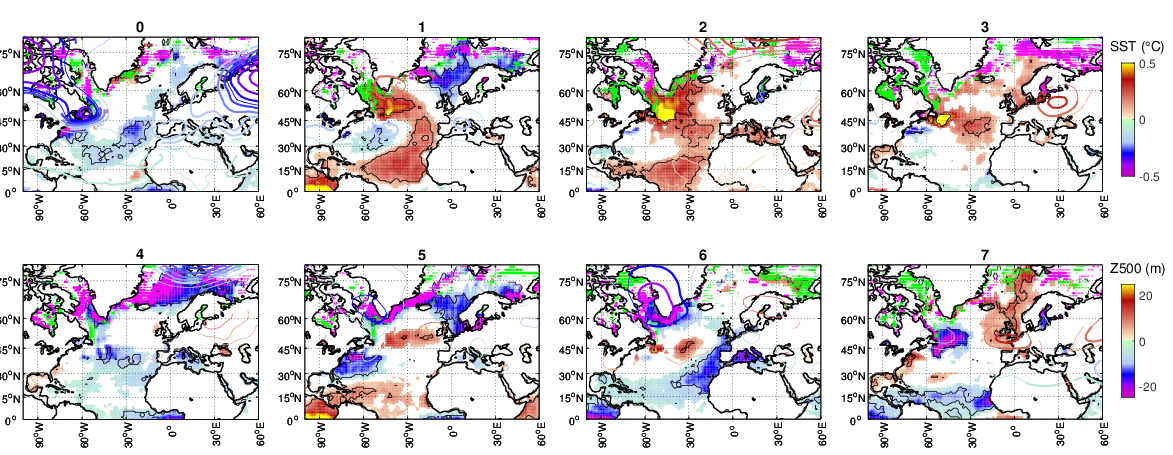


**Figure S11 -** Multiannual evolution of anomalous summer patterns around peak wet phases linked to decadal variability of Po River discharges. Shading: summer (AS) sea-surface temperatures, only for significant signals at 75% confidence (black contours identify region significant at 90% confidence); dots: September sea ice concentration, only for local increases (magenta) and decreases (green) significant at 75% (small) and 90% (large) confidence; contour: 500 hPa AS geopotential height, only for significant values at 75% (thin) and 90% (thick) confidence. Z500 data are linearly detrended, sea-surface temperature and sea-ice data are detrended with a second order polynomial fit to the data. Results are for years {1902 1914 1936 1950 1964 1978 1996} accounting for uncertain sampling. Panel titles indicate the lag, in years, from the reference years. Sea ice results are only indicative, given the more limited temporal coverage of the dataset.


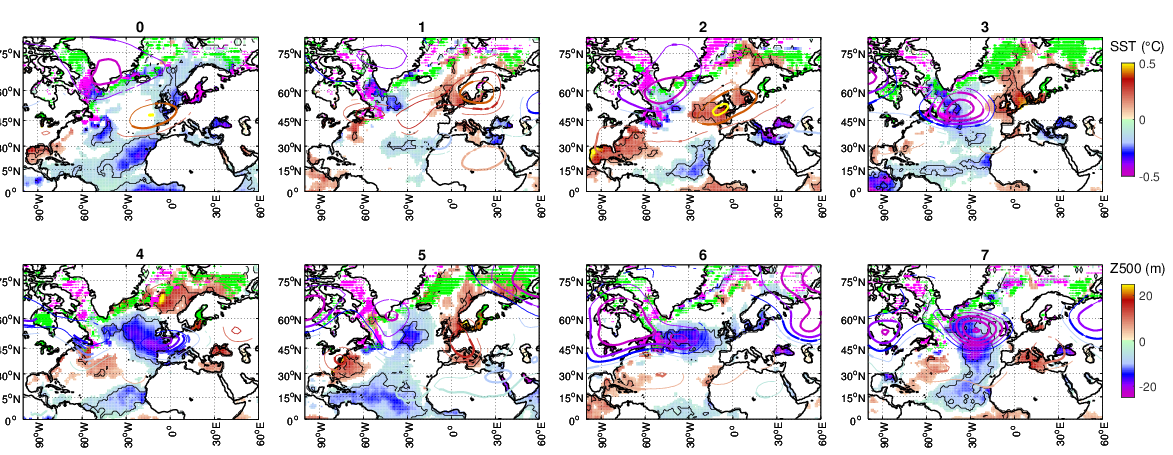


**Figure S12 -** Multiannual evolution of anomalous winter patterns around peak dry phases linked to decadal variability of Po River discharges. Shading: winter (OM) sea-surface temperatures, only for significant signals at 75% confidence (black contours identify region significant at 90% confidence); dots: March sea ice concentration, only for local increases (magenta) and decreases (green) significant at 75% (small) and 90% (large) confidence; contour: 500 hPa OM geopotential height, only for significant values at 75% (thin) and 90% (thick) confidence. Z500 data are linearly detrended, sea-surface temperature and sea-ice data are detrended with a second order polynomial fit to the data. Results are for years { 1907 1920 1943 1957 1971 1987} accounting for uncertain sampling. Panel titles indicate the lag, in years, from the reference years. Sea ice results are only indicative, given the more limited temporal coverage of the dataset.


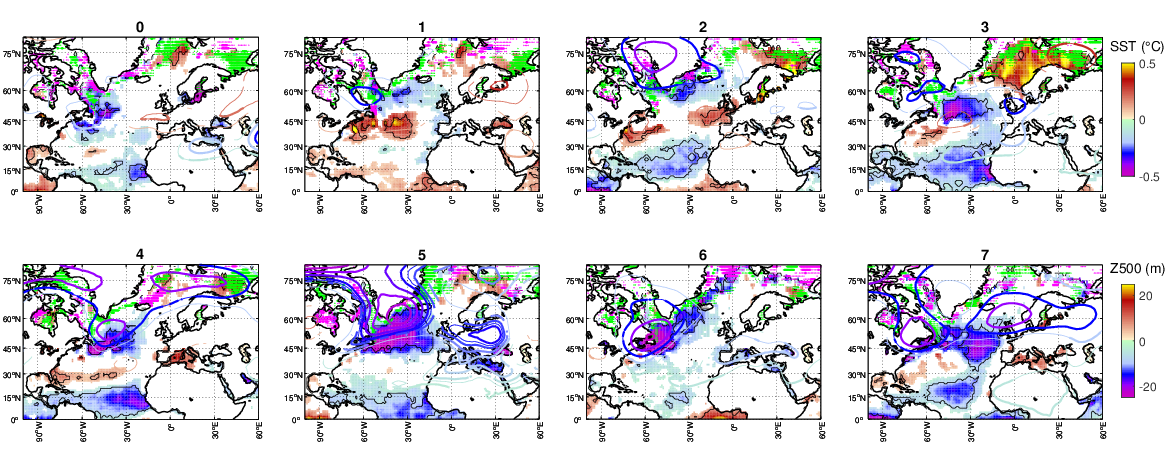


**Figure S13 -** Multiannual evolution of anomalous summer patterns around peak dry phases linked to decadal variability of Po River discharges. Shading: summer (AS) sea-surface temperatures, only for significant signals at 75% confidence (black contours identify region significant at 90% confidence); dots: September sea ice concentration, only for local increases (magenta) and decreases (green) significant at 75% (small) and 90% (large) confidence; contour: 500 hPa AS geopotential height, only for significant values at 75% (thin) and 90% (thick) confidence. Z500 data are linearly detrended, sea-surface temperature and sea-ice data are detrended with a second order polynomial fit to the data. Results are for years { 1907 1920 1943 1957 1971 1987} accounting for uncertain sampling. Panel titles indicate the lag, in years, from the reference years. Sea ice results are only indicative, given the more limited temporal coverage of the dataset.


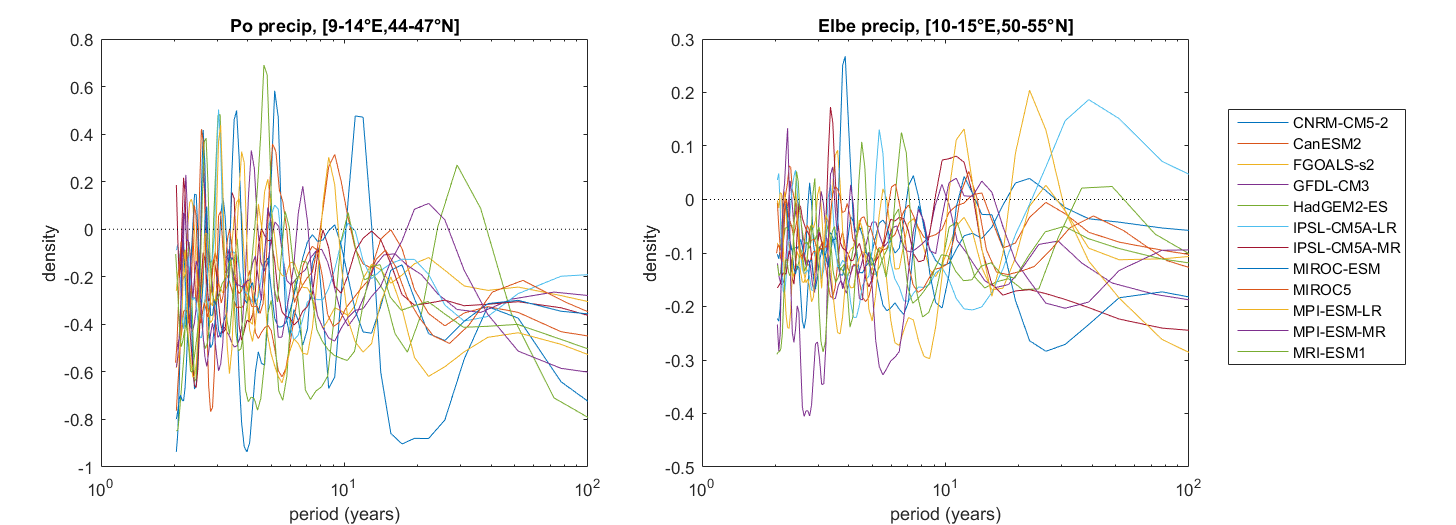


**Supplementary Figure S14 –** Density spectra of winter (October-March) regional precipitation simulated by a multi-model ensemble of historical simulations from the Coupled Model Intercomparison phase 5 database. Spectra are plotted relative to their 95% confidence level (horizontal black dotted line). Note that the different simulations cover different periods, which affects the comparability of the spectra. All simulations are the realization number 1 of the historical ensemble of the respective model.


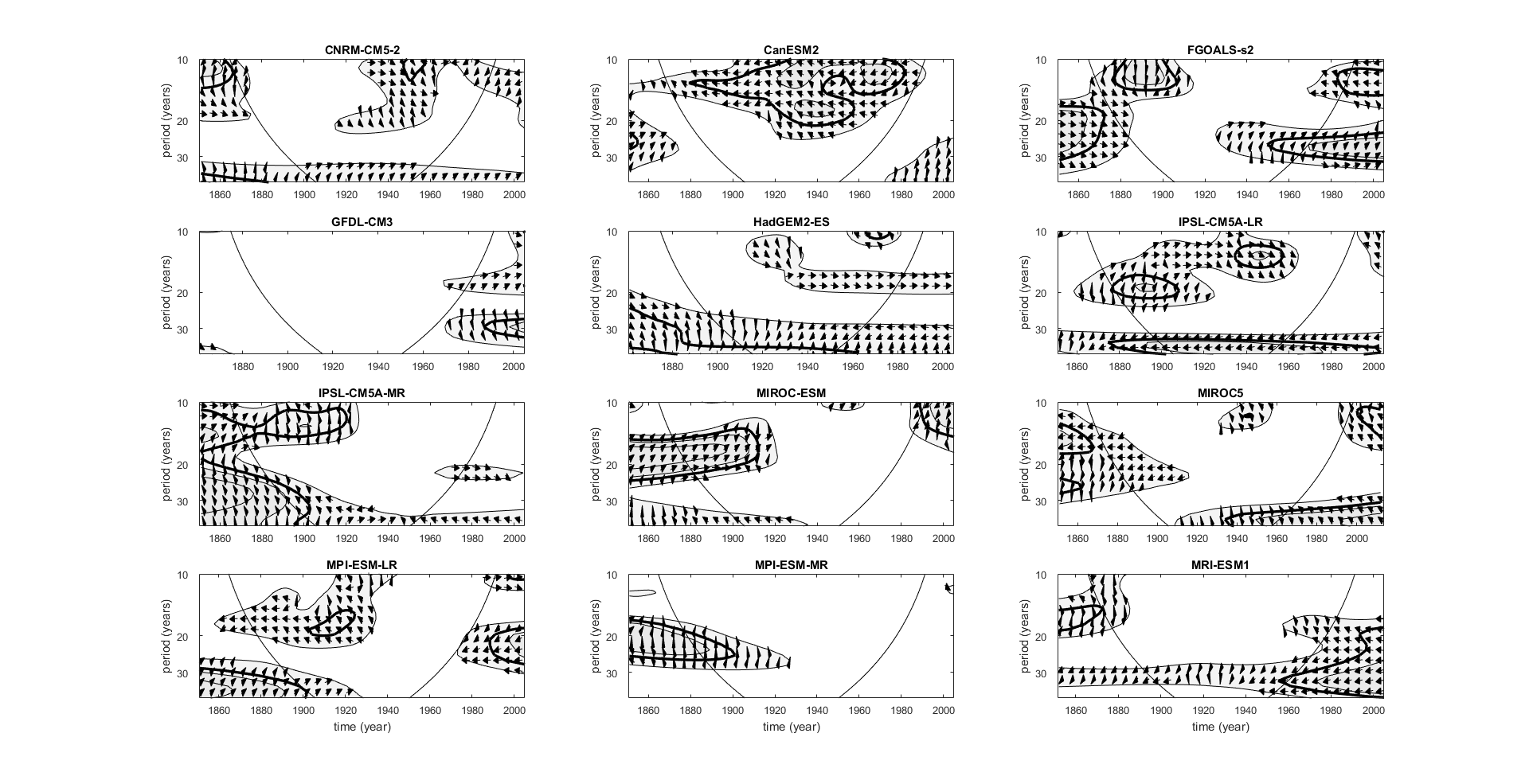


**Supplementary Figure S15** – Wavelet coherence spectra between winter-average (October-March) precipitation spatially averaged over the Po and Elbe river basins simulated by a multi-model ensemble of historical simulations from the Coupled Model Intercomparison phase 5 database. Spatial averages are calculated over the domain 9-14°E, 44-47°N for the Po, and 10-15°E, 50-55°N for the Elbe. Thick contours identify the 5% significance level. Arrows identify the phases (eastward-pointed arrows identify co-phase, Po discharges lead for clockwise rotation from the co-phase axis). The shaded region indicates where edge effects occur.

**Supplementary Table S1** – Long-term trend analysis: results of linear regressions of time (years y) on European river discharges (d) d = β + αy, for the annual, winter and summer averages. Trends significant at the 5% level are highlighted with bold fonts.

**Annual average**

**River (period of analysis) α statistics Estimate SE tStat pValue**

Po Pontelagoscuro (1900-2015): -0.74268 1.0324 -0.71936 0.47339

Danube Bratislava (1901-2007): 0.30913 1.0245 0.30173 0.76345

Elbe Decin (1900-2014): 0.028778 0.2668 0.10786 0.9143

Rhine Duesseldorf (1931-2012): 2.5124 2.1369 1.1757 0.24319

Rhine Rees (1900-2012): 1.721 1.3172 1.3066 0.19404

Rhine Basel (1900-2014): 0.1506 0.48172 0.31264 0.75513

Rhine Koeln (1900-2012): 1.7262 1.2203 1.4146 0.15999

Rhone Chancy (1904-2014): -0.041695 0.14998 -0.278 0.78154

Rhone Porte Du Scex (1905-2014): 0.094733 0.063768 1.4856 0.14031

Loire Montjean (1900-1979): 0.14513 1.441 0.10071 0.92004

Oder Gozdowice (1901-1993): -0.085558 0.535 -0.15992 0.8733

Oder Hohensaaten (1946-2012): 0.14941 0.79913 0.18696 0.85227

Dniestr Tighina (1945-1985): **3.3552 1.1876 2.8252 0.0074102**

Wisla/Vistula Tczew (1901-1993): 0.43527 0.9052 0.48085 0.63178

**Winter average**

**River (period of analysis) α statistics Estimate SE tStat pValue**

Po Pontelagoscuro (1900-2015): 1.1134 1.4486 0.76864 0.4437

Danube Bratislava (1902-2008): 2.1236 1.1249 1.8878 0.061816

Elbe Decin (1900-2014): 0.036649 0.35948 0.10195 0.91898

Rhine Duesseldorf (1932-2012): 4.7306 2.9564 1.6001 0.11356

Rhine Rees (1900-2012): 3.1002 1.8174 1.7059 0.090831

Rhine Basel (1900-2014): **1.4068 0.51602 2.7262 0.0074288**

Rhine Koeln (1900-2013): **3.417 1.6434 2.0792 0.039882**

Rhone Chancy (1905-2014): **0.7876 0.15907 4.9513 2.7369e-06**

Rhone Porte Du Scex (1906-2014): **0.76447 0.043448 17.595 0**

Loire Montjean (1900-1979): 0.63275 2.2227 0.28467 0.77665

Oder Gozdowice (1902-1994): -0.30464 0.74513 -0.40884 0.68362

Oder Hohensaaten (1947-2013): 0.50856 1.0835 0.46937 0.64038

Dniestr Tighina (1946-1985): 2.5277 1.2823 1.9712 0.056021

Wisla/Vistula Tczew (1902-1994): 0.75968 1.2557 0.60498 0.5467

**Summer average**

**River (period of analysis) α statistics Estimate SE tStat pValue**

Po Pontelagoscuro (1900-2015): -2.2071 1.2548 -1.759 0.081267

Danube Bratislava (1901-2008): -1.7281 1.3886 -1.2445 0.21607

Elbe Decin (1900-2014): 0.039515 0.30288 0.13046 0.89643

Rhine Duesseldorf (1931-2012): 0.45821 2.1923 0.20901 0.83497

Rhine Rees (1900-2012): 0.38577 1.3773 0.28009 0.77993

Rhine Basel (1900-2014): -1.0536 0.63574 -1.6572 0.10025

Rhine Koeln (1900-2013): 0.40243 1.2643 0.31831 0.75084

Rhone Chancy (1904-2014): **-0.84972 0.20916 -4.0626 9.1761e-05**

Rhone Porte Du Scex (1905-2014): **-0.57471 0.11133 -5.1621 1.1206e-06**

Loire Montjean (1900-1979): -0.16311 1.2192 -0.13379 0.89392

Oder Gozdowice (1901-1994): 0.12394 0.57997 0.21371 0.83125

Oder Hohensaaten (1946-2013): 0.40255 0.88651 0.45409 0.65125

Dniestr Tighina (1945-1985): **4.5956 1.8571 2.4746 0.017795**

Wisla/Vistula Tczew (1901-1994): -0.0024215 1.1582 -0.0020907 0.99834
